# Depression Prediction by Using Ecological Momentary Assessment, Actiwatch Data, and Machine Learning: Observational Study on Older Adults Living Alone

**DOI:** 10.2196/14149

**Published:** 2019-10-16

**Authors:** Heejung Kim, SungHee Lee, SangEun Lee, Soyun Hong, HeeJae Kang, Namhee Kim

**Affiliations:** 1 College of Nursing Yonsei University Seoul Republic of Korea; 2 Mo-Im Kim Nursing Research Institute Yonsei University Seoul Republic of Korea; 3 BRFrame Inc Seoul Republic of Korea; 4 Health-IT Acceleration Platform Technology Innovation Center College of Medicine Yonsei University Health System Seoul Republic of Korea

**Keywords:** elderly, one-person household, depression, ecological momentary assessment, actigraphy, machine learning

## Abstract

**Background:**

Although geriatric depression is prevalent, diagnosis using self-reporting instruments has limitations when measuring the depressed mood of older adults in a community setting. Ecological momentary assessment (EMA) by using wearable devices could be used to collect data to classify older adults into depression groups.

**Objective:**

The objective of this study was to develop a machine learning algorithm to predict the classification of depression groups among older adults living alone. We focused on utilizing diverse data collected through a survey, an Actiwatch, and an EMA report related to depression.

**Methods:**

The prediction model using machine learning was developed in 4 steps: (1) data collection, (2) data processing and representation, (3) data modeling (feature engineering and selection), and (4) training and validation to test the prediction model. Older adults (N=47), living alone in community settings, completed an EMA to report depressed moods 4 times a day for 2 weeks between May 2017 and January 2018. Participants wore an Actiwatch that measured their activity and ambient light exposure every 30 seconds for 2 weeks. At baseline and the end of the 2-week observation, depressive symptoms were assessed using the Korean versions of the Short Geriatric Depression Scale (SGDS-K) and the Hamilton Depression Rating Scale (K-HDRS). Conventional classification based on binary logistic regression was built and compared with 4 machine learning models (the logit, decision tree, boosted trees, and random forest models).

**Results:**

On the basis of the SGDS-K and K-HDRS, 38% (18/47) of the participants were classified into the probable depression group. They reported significantly lower scores of normal mood and physical activity and higher levels of white and red, green, and blue (RGB) light exposures at different degrees of various 4-hour time frames (all *P*<.05). Sleep efficiency was chosen for modeling through feature selection. Comparing diverse combinations of the selected variables, daily mean EMA score, daily mean activity level, white and RGB light at 4:00 pm to 8:00 pm exposure, and daily sleep efficiency were selected for modeling. Conventional classification based on binary logistic regression had a good model fit (accuracy: 0.705; precision: 0.770; specificity: 0.859; and area under receiver operating characteristic curve or AUC: 0.754). Among the 4 machine learning models, the logit model had the best fit compared with the others (accuracy: 0.910; precision: 0.929; specificity: 0.940; and AUC: 0.960).

**Conclusions:**

This study provides preliminary evidence for developing a machine learning program to predict the classification of depression groups in older adults living alone. Clinicians should consider using this method to identify underdiagnosed subgroups and monitor daily progression regarding treatment or therapeutic intervention in the community setting. Furthermore, more efforts are needed for researchers and clinicians to diversify data collection methods by using a survey, EMA, and a sensor.

## Introduction

### Challenges of Geriatric Depression

Depression is one of the most prevalent mental health problems in older adults. Globally, it was the third leading cause of years lived with disability in 2015, increasing by 18.4% between 2005 and 2015 [1]. In particular, older adults living alone are most vulnerable to depression compared with those living with others [2,3] because 30.2% of Koreans living alone are reported to have significant symptoms of depression [4]. In Korea, the economic burden of individuals with depression increased by about 27.1% from 2009 to 2013, with an estimated total cost of Korean $27.1 billion [5]. Thus, early and accurate diagnosis is critical to initiate timely treatment and reduce further disease burden; however, it is difficult to diagnose geriatric depression [1,2].

Underdiagnosis or inaccurate diagnosis of depression in older adults living alone remains a challenge [2]. Self-report and clinical interview are the primary methods to diagnose depression [6]; however, there are several challenges to overcome when diagnosing older adults living alone. First, no proxy currently exists that uses objective observations for reporting older adults’ depressive symptoms in their daily lives. Second, existing self-reporting instruments have a limitation to assess mood variability in response to daily stress in the natural environment, even the validity and reliability [6]. Third, atypical symptoms of depression are more common in older adults compared with younger adults [2,6,7]. Finally, older Asian adults are often hesitant to report their depressed mood and depressive symptoms because of social stigma or misconceptions [8]. Thus, it is vital to use diverse methods to collect additional data to fill the gaps contributing to the underdiagnosis of geriatric depression [2,6].

### Potential Methods to Identify Geriatric Depression

Ecological momentary assessment (EMA) has been suggested as a promising instrument because it can detect an individual’s real-time experiences and mood in real-world settings over time and in different situations [9]. Currently, clinicians rely on the retrospective reports of patients’ depressed mood in unfamiliar examination rooms using standardized instruments [6,10]. However, EMA allows individuals to report their momentary mood in the *right now and here*, multiple times in their real and familiar environment [9,11], rather than in artificial settings or laboratories. Thus, health care professionals can collect high ecologically valid data that are more specific to the older adult’s contextual situation and readily applicable to lifestyle modification [9,11,12].

As some older adults have difficulty using high-tech devices, one of the challenges with using EMA by older adults is the limited choices available when choosing a device [9]. Although a diverse number of smart devices are available, a wrist-worn Actiwatch is one method for collecting ecological momentary data [11] and other types of data. It has been primarily used to measure objective sleep and motor activity [13,14]. The usefulness of actigraphy has been well recognized and validated in individuals with depression [15] because of its familiarity, portability, and simple operation that involves pressing the device button to input the data [13]. Although actigraph has been suggested as being an advantageous device for use with older adults [16], few studies have used actigraphy to measure momentary mood or related problems in older adults with depression [17,18].

EMA and actigraphy data require a new analytic approach rather than conventional analysis. Numerous studies have used machine learning with wearable sensor data to improve symptom detection and for monitoring in diverse populations of neuropsychiatric patients living in real-time and in diverse life contexts [19-21]. Specifically, the assessment of suicide risk and emotional distress was identified as a possibly applicable area for using social media text mining [22], predicting negative emotions using mobile phone usage pattern [23], and detecting everyday behavior related to clinically meaningful levels of depression using sensor data [24]. Machine learning involves an objective, data-driven, and situation-independent analysis, and it has also been applied in the analysis of sensor data for automated assessment of mental health [24-26]. Machine learning analytic techniques that computationally mine meaning from data and classify, detect, and segment meaningful patterns, associations, relationships, and trends between variables and simulation are used to build predictive and optimization models [25,27]. These data analytics can equally be applied to small data to extract and model insights [25,27]. Therefore, depression is an ideal construct to establish, validate, and clinically apply machine learning approaches using sensor data. Thus, this study aimed to develop a supervised machine learning algorithm to predict the classification of depression groups among older adults living alone using EMA data from older adults living with a depressed mood.

## Methods

### Overview of Study Design

**Figure 1:**
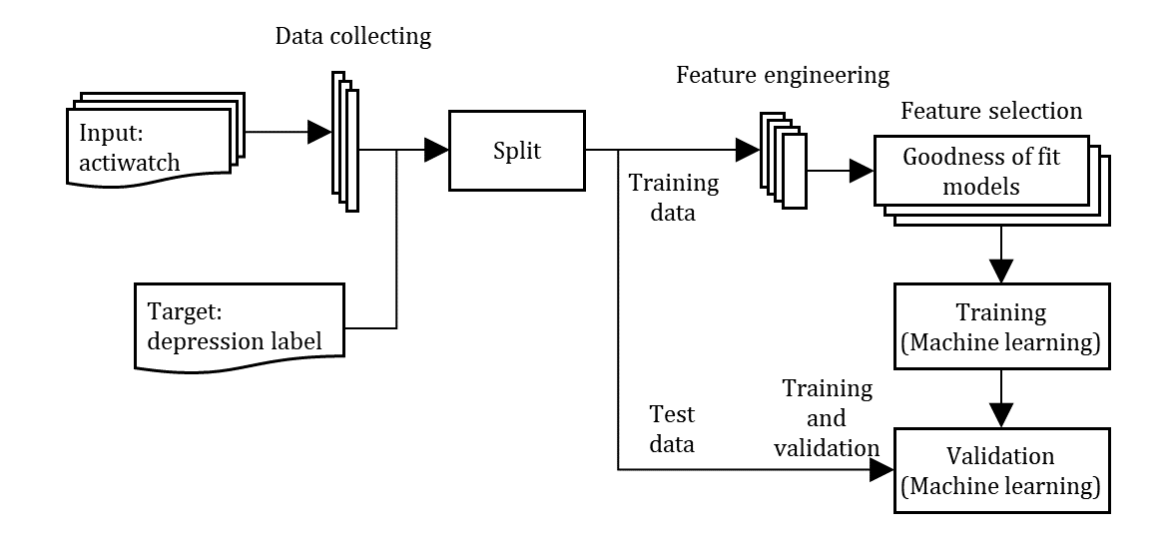
Construction prediction model.

On the basis of expert opinion [28] and a methodological guide [27], this study was conducted in 4 steps: (1) collecting data associated with depression, (2) data processing and representation, (3) data modeling (ie, feature engineering and selection), and (4) training and validation of the prediction model (Figure 1).

### Data Collection

#### Sample

We recruited a convenience sample of 56 older adults living alone via a community health center in Korea between May 2017 and January 2018. Inclusion criteria were as follows: the participants (1) were aged 65 to 94 years, (2) understood Korean, (3) lived as a single household in the community, and (4) had at least mild levels of depressive symptoms (score ≥5 on the Short Geriatric Depression Scale). We excluded 3 individuals with significant cognitive impairment and a high risk of suicide according to the Korean versions of the Mini-Mental Status Examination [29] and Crisis Triage Ration Scale [30]. In addition, the data from 6 individuals were excluded from the analysis because of refusal to wear or incomplete wearing of an Actiwatch (n=5) and data loss because of device error (n=1). Thus, the final sample comprised 47 participants who constantly wore an Actiwatch and reported sufficient numbers of EMA (ie, at least once a day) for 2 weeks [31]. All participants provided written informed consent, and the institutional review board of the affiliated university (IRB 2017-0007-1) approved the study.

#### Depression Measures

To classify the depression and nondepression groups, the Korean versions of the Short Geriatric Depression Scale (SGDS-K) and the Hamilton Depression Rating Scale (K-HDRS) were used to assess self-reported and clinician-observed symptoms of depression at baseline and 2 weeks. The SGDS-K is a 15-item instrument to assess subjective depressive symptoms (0=symptom absent and 1=symptom present). The total score ranges from 0 to 15; a higher score indicates more severe levels of depression [32]. The K-HDRS is a 17-item instrument that is the most widely used observer rating scale for observing depressive symptoms and severity. The total score ranges from 0 to 52, and a higher score indicates more severe depression [33]. The diagnostic validity of SGDS-K and K-HDRS for screening has been reported in previous studies [33,34].

#### Actigraphy Data and Ecological Momentary Assessment

Physical activity and ambient light exposure were measured using a wrist-worn Actiwatch (Actiwatch Spectrum PRO, Philips Respironics). It is considered to be a valid and reliable accelerometer that continually detects wrist movements that reflect activities; sleep-wake patterns; light exposure to the red, green, and blue (RGB) spectral regions; and broad-spectrum white light [35,36]. Data were collected continuously in 30-second epochs for 14 consecutive days. Participants wore the Actiwatch on the nondominant wrist all the time and were instructed to take it off only when taking a bath or for a few minutes as needed. Furthermore, participants were instructed that long sleeves should not cover the light sensor in the Actiwatch. We used Actiware software (Philips Respironics) to export the data.

Participants were instructed to rate their momentary mood using a button on the Actiwatch for 2 weeks. When the participants pressed the button, an increasing number was shown in the front window of the Actiwatch. The mood level was measured on a 10-point Likert scale (ranging from 1=very depressed to 10=not depressed) on the individualized preset time, as EMA reporting time in a day should be set in consideration of each individual’s lifestyle and convenience [37]. The Actiwatch included options for audible and vibrating alarms that were set to appear 4 times a day as a means to remind participants to complete the EMA regarding their current depressed mood. When a participant missed the alarm to complete the EMA, they received additional alarms over 5-min intervals until they responded. Research staff also provided a follow-up phone call to all participants after 1 week, and additional contact was provided for troubleshooting on an *as-needed* basis. Participants received gifts that were worth US $10 for completing the 2-week data collection.

### Data Processing and Representation

#### Defining Depression

**Figure 2:**
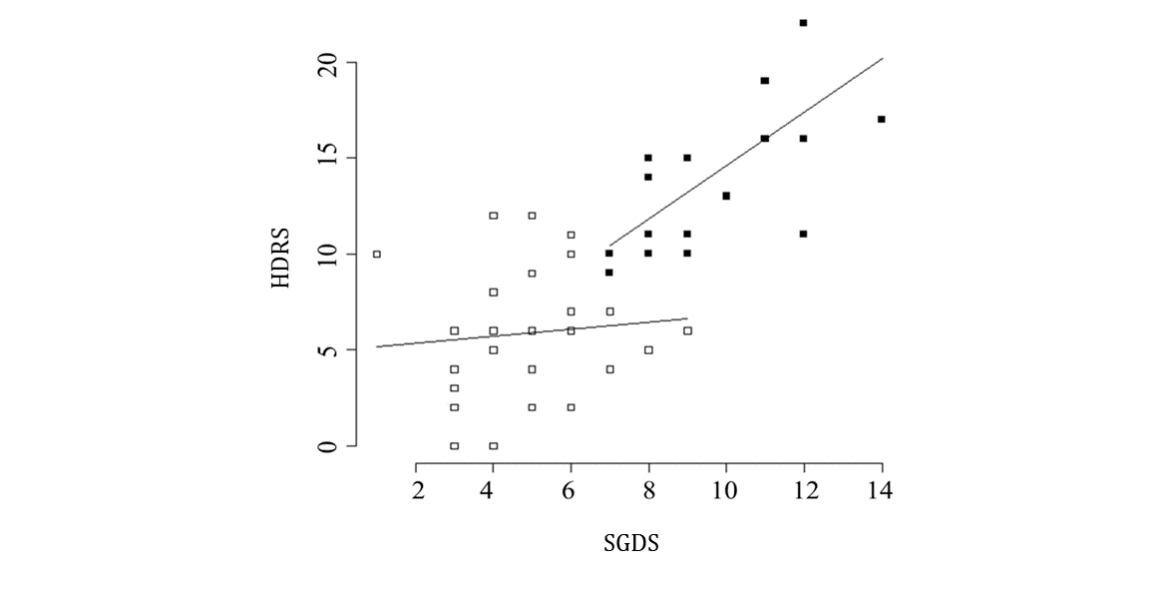
Correlations between Short Geriatric Depression Scale (SGDS) and Hamilton Depression Rating Scale (HDRS) scores by the depression groups. White squares mean nondepression group, whereas the black squares mean depression group.

The diagnosis of depression in older adults living alone was made using a decision-making model that is primarily used in the machine learning method [38]. To assess depression, SGDS-K and K-HDRS were administered at baseline and 2 weeks later. The data for a probable diagnosis of depression were based on the second measure at 2 weeks, during which the correlation between SGDS-K and K-HDRS was the highest (r=0.753; *P*=.01). To develop a diagnostic model, participants were classified into 2 groups: depression (SGDS-K≥7 and K-HDRS≥8) and nondepression (SGDS-K≤6 and K-HDRS≤7) groups [39-41]. Finally, 18 older adults were classified into the depression group and 29 were classified into the nondepression group (Figure 2).

#### Data Processing

Data were collected through the Actiwatch for 2 weeks. Data preprocessing began with the downloading of raw data and conversion into usable data. In a participant’s activity time series, continuous data were collected by the Actiwatch every 30 seconds. Triaxial data were calculated as an activity count. On the basis of the characteristics of signal data collected in the time series at 30-second intervals, null or abnormal values because of user or device error were checked and excluded from the analysis.

If a participant removed the device, the triaxial accelerometer would record values of 0 to indicate the duration of time for which it was not worn. Natural human behavior involves micromovements that are sensed by the accelerometer even during sleep, and therefore, periods with a continuous absence of movement indicate device removal. The raw accelerometer data were aggregated into minute-by-minute epochs using a script written in R (version 3.3.1). Data were considered as missing or being excluded from the analysis when (1) there were 0 measures in 5 min despite the indication of the *wearing* status or (2) more than 60% of a participant’s data were missing in a day.

The processing of EMA scores was performed as follows: (1) exploring the pattern of each participant’s EMA scores of depressed mood at 4 different time points each day, (2) comparing the 4 EMA scores and the grand mean scores between 2 groups, (3) examining the overall patterns between 2 groups, and (4) computing a composite score of daily EMA score.

### Data Modeling of Depression Prediction

#### Feature Engineering

Feature engineering is a step of parameterizing the collected raw data. It is a data mining process that quantitatively and qualitatively characterizes the statistics of the measured values for each participant through data aggregation or pattern analysis [38]. Moreover, it is important to determine additional variables to consider for the prediction model during feature engineering [38]. The variables to be included in the prediction model are the original variables included in the existing data and the new types of variables created using these primitive variables. Through the feature engineering process, the existing and new variables are identified to specify the characteristics of the participants and improve the accuracy of the prediction models [38].

After examining the daily fluctuations in the EMA, activity, and ambient light exposure, we compared both groups’ 4-hour mean differences in the selected variables using a Mann-Whitney U test and time series plot. To find additional variables to consider for the prediction model, we tested diverse types of sleep parameters, such as total time in bed, total sleep time, sleep efficiency, and wake-after-sleep-onset (WASO).

#### Feature Selection

This process selects and filters significant variables used in the prediction model. Owing to the small sample size, unnecessary variables in the process of predicting depression may decrease the model’s degree of freedom, which can negatively affect the explanatory power or the normal operation of the model [38]. Therefore, it is necessary to evaluate the significance of the processed variables through hypothesis testing and logistic regression analysis to select the most efficient combination of variables [38].

The actual selection of the variables was performed by a mean difference test of the statistics of the depression and nondepression groups and by logistic regression analysis based on various combinations of variables to examine the fitness of the models and determine the final combination of explanatory variables to be used in the prediction [38]. We selected EMA score, activity, and ambient light exposure and sleep efficiency to perform a binary logistic regression.

### Training and Validation Test

We evaluated training and validation data of the predictive model to classify depression groups based on the previously selected explanatory variables through the cross-validation process and assessed the validity of the final model [38]. Data from 47 participants were divided into training data and test data, and the machine learning method was applied to calculate the predictive power of the model. Specifically, the parameter value of the prediction model was calculated from the learning data and was applied to the test data, and the prediction level was evaluated using rxLogit function in Microsoft R package.

#### Data Partitioning

**Figure 3:**
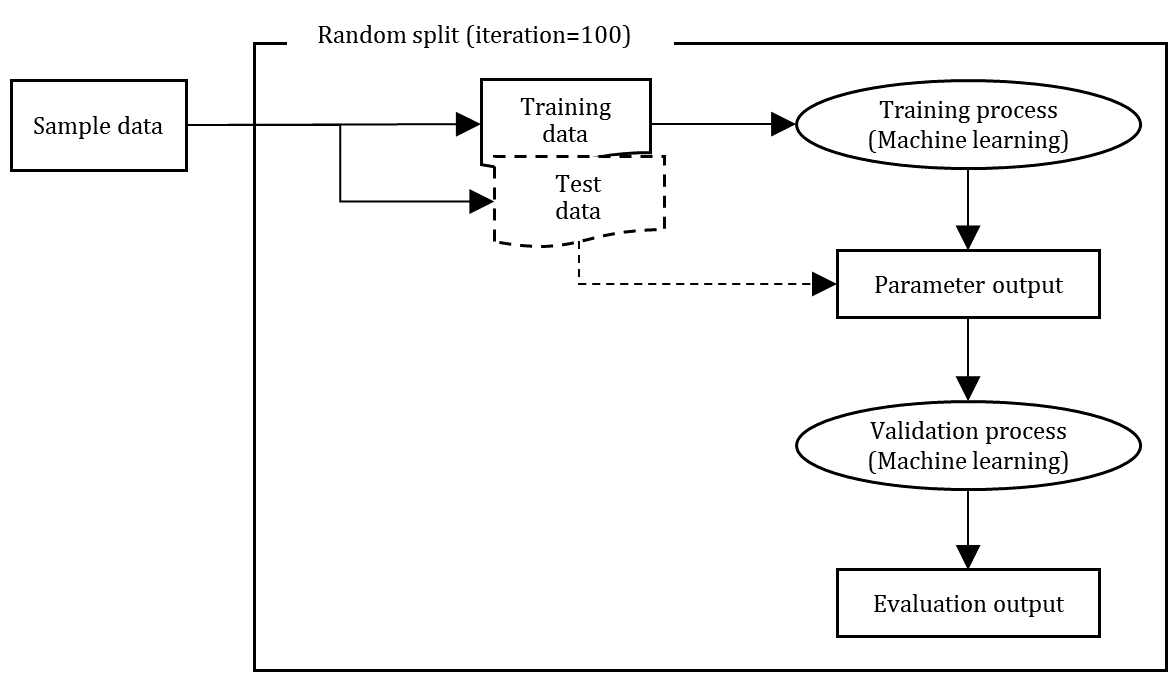
Machine learning—training, validation, and test.

To train our models without overfitting and test their subsequent performance, we randomly partitioned the dataset. Each time series was randomly assigned to a partition while maintaining an even class distribution of the target variable, depression group. Data were split at a 0.65:0.35 ratio for training and testing, respectively. Thus, the data were divided as follows: 30 as training data (n=20 for the nondepression group and n=10 for the depression group) and 17 as test data (n=9 for the nondepression group and n=8 for the depression group) in consideration of the sample size. Ideally, to ensure the cross-validation of the model and the validity of the evaluation, the data resplitting process should be repeated 100 times or more at random (Figure 3).

#### Training the Models

Machine learning techniques that were used for training and validation included the logit, decision tree, boosted trees, and random forest models. To test the validity of the prediction model, several indices were used such as accuracy, precision, recall (or sensitivity), specificity, F score, and area under receiver operating characteristic curve (AUC) in comparison between the machine learning model and a logistic regression, which are mainly used in the classification model [38]. Each indicator was calculated in the Confusion matrix. Our model showed that depression was positive and nondepression was negative.

## Results

### Descriptive Statistics of the Sample

The average age of the 47 participants was 78 (SD 5.24) years; they were mainly women (44/47, 94%), had less than high school education (42/47, 89%), and had a moderate socioeconomic status (35/47, 74%). The sample characteristics of the 6 participants who were excluded from the analyses did not significantly differ from those of the 47 participants included in the final analyses.

On the basis of the traditional depression assessment tools (SGDS-K and K-HDRS), 38% (18/47) of the participants were classified into the depression group. We performed the Mann-Whitney U test to compare the 2-week grand means of the depression and nondepression groups, and significant differences were identified. The depression group reported a significantly lower score of nondepressed mood and physical activity and higher levels of exposure to white and RGB light (all *P* values <.01). Data were used to identify the pattern of time series of signal data and for reference for interpretation (Table 1).

**Table 1:** Mean differences tested by the Mann-Whitney U test.

Characteristics		Nondepression group (n=29), mean (SD)	Depression group (n=18), mean (SD)	Differences, mean (SD)	*P* value
Ecological momentary assessment		6.6 (1.55)	5.1 (1.64)	−1.5 (1.58)	.004
Activity (counts)		90.5 (32.76)	67.4 (18.82)	−23.1 (28.31)	.003
**Light exposure (lux)**					
	White	54.0 (36.54)	81.5 (39.98)	27.5 (37.88)	.008
	Red	69.9 (54.12)	103.6 (63.95)	33.7 (58.03)	.03
	Green	60.1 (43.09)	99.9 (55.35)	39.8 (48.09)	.005
	Blue	38.9 (28.84)	62.0 (34.33)	23.1 (31.03)	.006

### Feature Engineering and Selection

#### Mean Differences in Different Sections of Time

To obtain a descriptive picture of the daily fluctuations in EMA, activity, and ambient light exposure and compare the 4-hour mean differences of the 2 groups, we performed the Mann-Whitney U test and time series plot. The depression group reported lower levels of EMA scores and daily activity throughout the day. However, there were higher levels of white and RGB light exposures in the depression group compared with the nondepression group.

**Figure 4:**
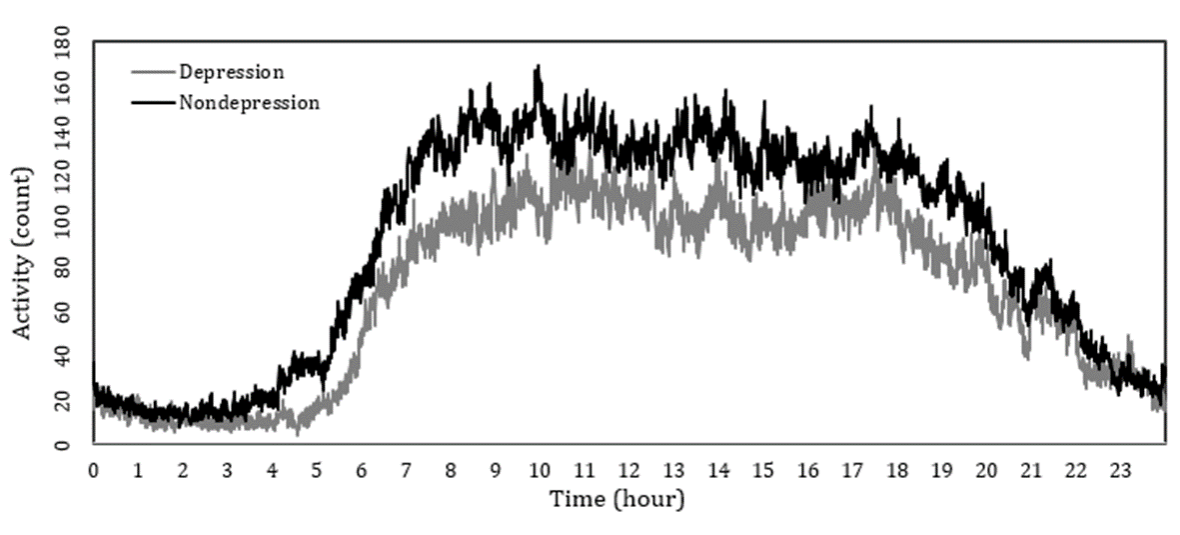
Daily time series plot of depression versus nondepression. Activity patterns for depression and nondepression groups. Y axis is average raw activity counts depending on each time of day and X axis is the time of day.

On further examination of different time frames, both groups showed the highest levels of activity in the morning between 8:00 am and 12:00 pm. However, the highest levels of white and RGB light exposures were observed between 12:00 pm and 4:00 pm in the nondepression group and 8:00 am and 12:00 pm in the depression group. More activities were observed for the depression group after 9:00 pm compared with the nondepression group, although the difference was not significant (Figure 4). This finding suggests that 4-hour data could be considered for modeling (Table 2).

**Table 2:** Mean differences in different sections of time.

Variables		12:00 am-4:00 am	4:00 am-8:00 am	8:00 am-12:00 pm	12:00 pm-4:00 pm	4:00 pm-8:00 pm	8:00 pm-12:00 am
**Ecological momentary assessment**							
	Nondepression	—^a^	6.4	6.6	6.6	6.6	—
	Depression	—	5.0	5.2	5.2	5.1	—
	Difference	—	−1.4	−1.4	−1.4	−1.5	—
	*P* value	—	.02	.009	.01	.005	—
**Activity**							
	Nondepression	16.4	78.5	141.2	133.0	120.1	54.1
	Depression	11.1	48.1	107.1	99.4	94.8	44.1
	Difference	−5.3	−30.4	−34.1	−33.6	−25.3	−10.0
	*P* value	.36	.02	.01	.005	.06	.20
**White light**							
	Nondepression	1.0	14.5	110.3	137.4	50.7	10.4
	Depression	0.7	18.1	196.3	161.6	91.7	20.6
	Difference	−0.3	3.6	86.0	24.2	41.0	10.2
	*P* value	.51	.42	.02	.08	.005	.10
**Red light**							
	Nondepression	0.6	17.1	150.0	181.7	64.2	5.8
	Depression	0.6	20.9	269.6	204.9	112.1	13.4
	Difference	0.0	3.8	119.6	23.2	47.9	7.6
	*P* value	.79	.43	.049	.12	.02	.07
**Green light**							
	Nondepression	1.1	17.0	125.0	154.2	54.3	8.9
	Depression	1.4	21.4	250.2	194.7	103.0	28.5
	Difference	0.3	4.4	125.2	40.5	48.7	19.6
	*P* value	.97	.31	.01	.05	.003	.09
**Blue light**							
	Nondepression	0.4	10.3	81.9	101.5	34.7	4.7
	Depression	0.4	13.7	154.6	126.2	64.8	12.3
	Difference	0.0	3.4	72.7	24.7	30.1	7.6
	*P* value	.77	.33	.01	.07	.006	.08

^a^Not applicable.

#### Additional Variable: Sleep Efficiency

As there were no differences in activity levels at night (see Figure 4) and no significant difference in other sleep components, sleep efficiency was chosen for modeling. Sleep efficiency is defined as the ratio of the total sleep time at night compared with the total amount of time spent in bed, which reflects night-time activity and the quality of sleep. We calculated sleep efficiency based on sleep time and WASO based on sleep component equations (Multimedia Appendix 1) [42].

In this study, activity for more than 5 consecutive minutes during the sleep time was considered as *wakefulness*. The total sum of all moments of wakefulness is referred to as WASO, when smoothing intermittent movements of night-time activity. A cutoff of 85% or higher was used to determine good sleep efficiency [42]. On the basis the mean differences in sleep efficiency of the 2 groups, the nondepression group had a sleep efficiency of 86% (25/29), which was higher than the 85% cutoff, whereas the depression group showed 83% (15/18), lower than the cutoff. The between group difference was different at the *P*=.08 level regarding sleep efficiency.

A binary logistic regression was performed to explore the best model fit using various combinations of variables (EMA score, activity, white and RGB light exposure, and sleep efficiency) depending on depression groups (nondepression group=0 and depression group=1). We compared diverse models using different means based on EMA and activity data. For example, we compared daily, morning-evening means, and all 4 scores as well as 4 different times of light exposure and other sleep components. Finally, we reached the conclusion that the variables of the final model were the best when using daily mean EMA score, daily mean activity level, white and RGB light at 4:00 pm to 8:00 pm exposure, and daily sleep efficiency. To consider collinearity between white and RGB light change and the small sample size, a computing score was calculated integrating means of white and RGB light between 4:00 pm and 8:00 pm. Table 3 shows goodness of fit depending on each variable. Our findings suggest that lower activity, higher EMA depressed mood, and exposure to white and RGB were associated with a higher likelihood of being classified as a depression group. Furthermore, this model showed a good model fit (accuracy: 0.705; precision: 0.770; specificity: 0.859; and AUC: 0.754) except 1 false-positive case.

**Table 3:** Estimates of logistic regression.

Variables, estimator		Estimates	*P* value
**Intercept**			.02
	Coefficient	35.200	
**Activity**			.02
	Coefficient	−0.095	
	Odds ratio (95% CI)	0.910 (0.84-0.98)	
	Marginal effect	−0.016	
**Computing score of white and red, green, and blue light**			.01
	Coefficient	0.024	
	Odds ratio (95% CI)	1.025 (1.01-1.04)	
	Marginal effect	0.004	
**Sleep efficiency**			.08
	Coefficient	−25.300	
	Odds ratio (95% CI)	<0.001 (<0.001-14.41)	
	Marginal effect	−4.362	
**Ecological momentary assessment**			.01
	Coefficient	−2.299	
	Odds ratio (95% CI)	0.100 (0.02-0.58)	
	Marginal effect	−0.397	

### Comparison of Four Machine Learning Methods

Table 4 shows the validation and test results for the prediction model based on machine learning. All variables included in the logistic regression were utilized (EMA score, activity, white and RGB light exposure, and sleep efficiency) to classify depression groups (nondepression group=0 and depression group=1). For cross-validation, bootstrapping with data partitioning and training models was performed 100 times. Among the 4 methods, the logistic regression model (accuracy: 0.910; precision: 0.929; and specificity: 0.940) had the best fit compared with the boosted trees and random forest models. Decision tree suffers from possible overestimated model fit because of sample size. boosted trees model showed relatively better fit compared with decision tree because of the modification process to correct errors through pruning. Random forest seemed less suitable for this study because of the small sample size and the limited number of tested variables [38].

**Table 4:** Evaluation metrics.

Evaluation metrics	Logistic regression		Decision tree		Boosted trees		Random forest	
	Mean (SD)	Minimum-maximum	Mean (SD)	Minimum-maximum	Mean (SD)	Minimum-maximum	Mean (SD)	Minimum-maximum
Accuracy	0.91 (0.007)	0.87-0.92	0.72 (0.03)	0.64-0.78	0.78 (0.04)	0.68-0.87	0.67 (0.05)	0.53-0.75
Precision	0.93 (0.01)	0.88-0.95	0.74 (0.04)	0.63-0.81	0.84 (0.06)	0.69-0.95	0.72 (0.11)	0.50-1.02
Recall/sensitivity	0.88 (0.01)	0.84-0.90	0.63 (0.05)	0.55-0.73	0.66 (0.06)	0.57-0.68	0.39 (0.07)	0.23-0.50
Specificity	0.94 (0.01)	0.90-0.96	0.80 (0.04)	0.72-0.88	0.84 (0.05)	0.78-0.98	0.88 (0.06)	0.80-1.04
F score	0.90 (0.01)	0.86-0.92	0.68 (0.03)	0.59-0.75	0.74 (0.05)	0.62-0.83	0.51 (0.05)	0.38-0.62
Area under receiver operating characteristic curve	0.96 (0.03)	0.91-0.99	0.66 (0.03)	0.61-0.71	0.84 (0.03)	0.79-0.89	0.71 (0.03)	0.69-0.79

## Discussion

### Principal Findings

The primary purpose of this study was to identify factors associated with geriatric depression in older adults living alone. We focused on developing a prediction model to classify the depression groups (probable depression vs nondepression) among older adults living alone. Along with the conventional instruments for depression screening, EMA of daily mood, actigraphy data of activity, and light exposure were utilized. Comparing diverse combinations of the selected variables, daily mean EMA score, daily mean activity, daily sleep efficiency, and exposure to white and RGB light between 4:00 pm and 8:00 pm for 2 weeks were selected for modeling. The cross-validation process was used to build a prediction model. Logit model showed compatible evaluation metrics compared with the traditional binary logistic regression. The use of both EMA and sensor data seems promising to develop a machine learning model for better identification of a probable depression among older adults [25].

### Comparison With Previous Studies

The depression group reported higher levels of daily depressed mood than did the nondepression group. In a small-scale study [17], the major depressive disorder (MDD) group reported higher levels of diurnal symptom patterns of negative affect with great variability than did the control group. Our study found that even the daily average of depressed mood was significantly related to the classification of depression groups determined by conventional screening [2,43]. This could help overcome a clinical challenge in diagnosing a psychiatric disorder during the first medical examination or interview by unfamiliar clinicians [2]. It can also be difficult for a person with a serious mental illness to complete the self-reporting questionnaire and for clinicians to make a quick diagnosis with limited time [6,44]. The findings of this study suggest that individuals with these difficulties may complete self-reports of depressed mood daily in their home environment. A simple question, although repetitive, might be more helpful to classify the probable depression groups as opposed to the application of complex and multiple-item questionnaires.

A significantly low level of daytime activity was observed in the depression group, similar to the findings of previous research [45]. A study [15] using actigraphy reported that patients with depression displayed less daytime motor activity than did individuals without depression. Related to the EMA of depression, high levels of momentary depressed mood had a lagged effect of prolonging the current status of being at home. Those who experience an increase in the average depressed mood the previous day tend to stay at home the following day [46]. Previous studies have reported that depressed people have decreased levels of daytime activity compared with healthy controls [2,15]. In adults aged over 60 years, depression is associated with lower levels of physical activity in global measure, compared with nondepressed individuals [47], similar to our study findings. In a meta-analysis of adults with MDD [48], more time was spent in sedentary behavior and less time in vigorous physical activities. Considering the relationship between physical activity and depression, promoting daytime physical activity could prevent the risk of depression in older adults [49].

Furthermore, sleep efficiency may be a potential factor to aid in classifying depression groups. Existing studies have confirmed the association between disrupted sleep and depression in adults [50-52] because sleep disturbance is one of the common misinterpreted symptoms of geriatric depression [6]. A study of older adults living alone found that depressed participants reported sleep-related issues [53]. However, our study did not clearly indicate that sleep efficiency was associated with the depression group because the sample size had low power to detect the significance (*P*<.08). Comparing with the findings of previous studies [12,54], this discrepancy should be interpreted with caution because the previous studies measured sleep components once through self-report, whereas our study assessed daily sleep efficiency for 2 weeks through repeated measures using actigraphy. Older adults living alone may have more difficulty in the early detection of sleep disturbances than do those living with others because of the absence of a bedroom partner [55]. Thus, further research using both subjective and objective measures is needed to confirm the predictive value of sleep efficiency with the larger sample.

Contrary to our expectation, higher levels of ambient light exposure were observed in the depression group, compared with the nondepression group. We expected that lower levels of ambient light exposure would be observed in the depression group compared with the nondepression group that had a less sedentary lifestyle and engaged in more outdoor activities [48]. There are limited explanations for this finding contrary to our hypothesis. A small-scale observational study [56] explored the subjective perception of lighting in depressed patients’ environment. When they were asked whether the lights in the surroundings seemed dimmer than usual, depressed patients answered that they perceived the environment to be dimmer than usual. Moreover, there was a significant association between depression severity and perception of dimness. Patients with severe (65%), moderate (21%), and mild (14%) depression responded that their ambient environment appeared dimmer than usual. To compensate for the perceived dim environment, they may turn on the lights at home most of the time or stay in places with more light. In addition, a depressed individual may turn on the lights to read books or do other things to compensate their awakening time at night. This behavior may affect heart rate variability, which is associated with mood regulation through ambient biofeedback [57]. Many relaxation methods or interventions use dimmed ambient lighting to soothe the patient [58], but individuals with depression may not prefer a dimmer environment that may increase the depressed mood.

### Implications for Geriatric Depression Research

Our study findings support 3 major inferences: (1) diversifying data collection methods to build an accurate model of depression prediction, (2) emphasizing the need to assess depressed mood at different time points in a day, and (3) monitoring multiple symptoms continuously. First, inactivity and poor sleep have been known to be symptoms of geriatric depression; however, the assessment of these symptoms is limited to using subjective reports of inactivity, sedentary lifestyle, or night-time sleep. Our study emphasizes the need for collecting both subjective and objective data related to mood disorders and examining intraday change patterns. For example, it is difficult to apply the Pittsburgh Sleep Quality Index global score to depressed older adults. Thus, clinicians should evaluate sleep efficiency through actigraphy as an objective measure, which may prevent the misinterpretation of a suspected depression [6]. Second, time-specific assessment is required to capture the significant features of specific data. Our study findings confirmed group differences in daytime activity, light exposure in the late afternoon, and night-time sleep. Thus, we suggest the need to collect data throughout the day and identify features closely related to depression [12,45]. Third, we suggest using sensor data, such as Actiwatch or activity tracking, for monitoring purpose. For example, strategies to improve physical activity should be included in the treatment for geriatric depression [48]. Monitoring daily activity of people with mood disorders using Actiwatch may help diagnose depression depending on the activity-based interventions [15]. In addition, our study shows some potential to use environmental data, such as ambient light, in identifying depression in the community setting. Sensor devices have a great potential to continuously capture diverse mental health–related information from the environment or context [24,25] in mental health.

### Implications of Electronic Health for Older Adults

EMA could be used as a diagnostic tool for depression in older adults. It has been used to assess multiple mental health indicators related to depressed mood [9]. In particular, older adults have less accurate retrospective memory, and therefore, the use of EMA for older adults can increase the accuracy of the assessment [10,59]. EMA has also been shown to have acceptability and feasibility for older adults, even those with cognitive and emotional difficulties [60]. In particular, EMA has the advantage of continuously reporting subjective symptoms according to the individual’s life patterns as digital phenotyping [44,61]. Thus, it is possible to collect personalized data, monitor the individual pattern, and examine fluctuations in the depressed mood within and between a day while reducing retrospective bias [10,11].

However, a limitation of this method is that multiple and repeated reports in a day are associated with a high dropout rate [11]. When applying EMA to older adults, sufficient device training and the practice of technical details could maximize the usability and appropriateness of EMA for older adults [60]. In our study, a dropout rate of 9% (5/53) was observed; thus, preventing dropout is important to ensure that older adults with depression complete multiple reports during the day after careful training and educating about the significance of EMA [11,16,62]. Selection of valid questions to measure momentary depressed mood and standardizing the EMA protocols are also important. Therefore, geropsychiatric clinicians should prepare to select the proper device, train older adults, and prevent dropout [16].

The paper-based diary approach has been traditionally used for older adults [6,63]; however, sometimes they do not accurately report their depressed mood because of response or retrospective bias or the social stigma associated with mental health illnesses [6,10,44,59]. Actigraphy is a noninvasive technique that provides simple and scientifically accurate data on a daily basis and can be used to improve health-related behaviors [64], thus suggesting its suitability for older adults. The 9% (5/53) dropout rate in our study indicates the acceptability and applicability of the Actiwatch for older adults, similar to a previous finding [11], especially as we identified a variety of subjective and objective factors that reflected an individual’s real-world environment, such as depressed mood, activity, sleep, and different light using a ubiquitous device.

Actigraphy also measures fluctuating subjective mood on a daily basis in real time and a natural environment through continuous recording [9] along with physical and environmental data [11]. An integrated approach to passive, objective, and continuous measurement has been used in psychiatry practice and research [44]. Moreover, passive actigraphy data and active EMA responses can be useful for understanding the characteristics of depression, offering insight into biological and environmental mechanisms underlying the depression, and developing individualized interventions for older adults [65]. Considering the rapid development of technologies, various types of sensor data may be used in geropsychiatric care for screening, monitoring, and diagnosing mental health [66-68]. Therefore, we expect that new measures, such as using sensor data, will help diagnose depression quickly and improve mental health problems.

### Limitations

Our study has several limitations. First, we used 2 screening instruments that relied on subjective reports to classify individuals in the 2 depression groups because these instruments had been widely used in community settings. However, a medical diagnosis based on Diagnostic and Statistical Manual of Mental Disorders–5 may be needed to define the clinically diagnosed depression group for data processing and representation [6]. In addition, further study may replicate our model including nondepressed older adults to establish screening models in general population. Although the EMA provides mood reports of older adults at the 4 time points every day for 2 weeks, our predictive model used daily grand mean that was relatively less frequent for EMA data collection. Replication studies that utilize data variability within a day should be tested over a longer time frame and use a more convenient mode to complete EMA to reduce subjective burden. Generalization of the EMA data and levels of activity is limited because every individual has different levels of normal with reference to a depressed mood or inactivity [6,11]. Thus, EMA with a real-life tagging system will be helpful to examine to what extent the data fluctuate from the average level for each individual. In addition, studies with larger samples are needed to ensure the generalizability of our findings considering the possible use of machine learning, which is an innovative method of feature extraction from data [21]. Specifically, the limited generalizability is expected for depressed men in older populations because the majority of older adults were women in Korea. Thus, it is necessary to oversample the groups of men for the next study on this topic [4].

### Conclusions

This study provides evidences to support the feasibility of machine learning in classifying depression groups based on EMA and actigraphy data. Specifically, our machine learning approach is applicable for females and elderlies with depressive mood when living alone in the community setting. The study findings provide 2 major inferences: (1) diversifying data collection methods to build an accurate model of prediction and (2) emphasizing the need for assessments at different time points in a day to obtain diverse data. Future researchers and clinicians should consider EMA and actigraphy to obtain data on daily mood, levels of activity and ambient light exposure, and sleep components in depressed older adults living alone.

## Appendix

Multimedia Appendix 1 Equation to calculate sleep efficiency based on sleep time and wake-after-sleep-onset.

## Abbreviations

AUC: area under receiver operating characteristic curve
EMA: ecological momentary assessment
K-HDRS: Korean version of the Hamilton Depression Rating Scale
MDD: major depressive disorder
NRF: National Research Foundation
RGB: red, green, and blue
SGDS-K: Korean version of the Short Geriatric Depression Scale
WASO: wake-after-sleep-onset
