# Supplementary material for: Depression Prediction by Using Ecological Momentary Assessment, Actiwatch Data, and Machine Learning: Observational Study on Older Adults Living Alone
Source: JMIR Mhealth Uhealth. 2019 Oct 16;7(10):e14149. doi: 10.2196/14149 (PMC6913579; doi:10.2196/14149)
Supplement: Multimedia Appendix 1 [file mhealth_v7i10e14149_app1.pdf]

$$\text{WASO} = \sum_{\substack{Sleep\\Onset\\Time}}^{\substack{Sleep\\Awakening\\Time}} \begin{cases} \| WakefulPeriod \|, & \| WakefulPeriod \| > 5, \\ 0 & otherwise \end{cases}$$

$$\text{Sleep Efficiency} = \frac{Total\ Sleep\ Time}{Total\ Minutes\ in\ Bed}$$

$$= \frac{\| Sleep\ Period \| - WASO}{\| Sleep\ Period \| + Latency}$$
